# Gram-Scale Synthesis of an Ultrastable Microporous Metal-Organic Framework for Efficient Adsorptive Separation of C_2_H_2_/CO_2_ and C_2_H_2_/CH_4_

**DOI:** 10.3390/molecules26175121

**Published:** 2021-08-24

**Authors:** Nuo Xu, Yunjia Jiang, Wanqi Sun, Jiahao Li, Lingyao Wang, Yujie Jin, Yuanbin Zhang, Dongmei Wang, Simon Duttwyler

**Affiliations:** 1 Key Laboratory of the Ministry of Education for Advanced Catalysis Materials, College of Chemistry and Life Sciences, Zhejiang Normal University, Jinhua 321004, China; Nuo_Xu@zjnu.edu.cn (N.X.); Yunjia_Jiang@zjnu.edu.cn (Y.J.); Wanqi_Sun@zjnu.edu.cn (W.S.); 17857171863@zjnu.edu.cn (J.L.); lywang@zjnu.edu.cn (L.W.); 2 Department of Chemistry, Yuquan Campus, Zhejiang University, 38 Zheda Road, Hangzhou 310027, China; 21837026@zju.edu.cn

**Keywords:** metal-organic frameworks, gas adsorption, C_2_H_2_/CO_2_ separation, C_2_H_2_/CH_4_ separation, gram-scale synthesis

## Abstract

A highly water and thermally stable metal-organic framework (MOF) Zn_2_(Pydc)(Ata)_2_ (**1**, H_2_Pydc = 3,5-pyridinedicarboxylic acid; HAta = 3-amino-1,2,4-triazole) was synthesized on a large scale using inexpensive commercially available ligands for efficient separation of C_2_H_2_ from CH_4_ and CO_2_. Compound **1** could take up 47.2 mL/g of C_2_H_2_ under ambient conditions but only 33.0 mL/g of CO_2_ and 19.1 mL/g of CH_4_. The calculated ideal absorbed solution theory (IAST) selectivities for equimolar C_2_H_2_/CO_2_ and C_2_H_2_/CH_4_ were 5.1 and 21.5, respectively, comparable to those many popular MOFs. The Q_st_ values for C_2_H_2_, CO_2_, and CH_4_ at a near-zero loading in **1** were 43.1, 32.1, and 22.5 kJ mol^−1^, respectively. The practical separation performance for C_2_H_2_/CO_2_ mixtures was further confirmed by column breakthrough experiments.

## 1. Introduction

Acetylene (C_2_H_2_) is one of most important fundamental chemicals in the petrochemical and electronic industries and is mainly produced by the cracking of petroleum or oxidative coupling of methane [1]. During such processes, some impurities like carbon dioxide (CO_2_) and methane (CH_4_) are cogenerated with C_2_H_2_ and must be removed to improve the quality of the C_2_H_2_ [2,3,4]. The traditional approaches for the separation of C_2_H_2_/CO_2_ and C_2_H_2_/CH_4_ based on cryogenic distillation or solvent extraction are either of high cost-/energy consumption or associated with pollution [5]. In this context, physisorptive separation using porous solid adsorbents has attracted particular interest due to the lower cost and energy penalty [6]. Among the diverse porous solid materials, metal-organic frameworks (MOFs) are of particular interest for such demands.

In the past decades, a large number of MOFs have been synthesized for various applications including but not limited to gas separation [7,8,9,10,11,12,13,14,15,16,17], carbon capture [18,19,20,21,22], pollutant removal [23,24], catalysis [25,26], sensing [27,28], energy devices [29,30], and water harvesting [31]. While many MOFs have shown remarkable separation selectivity for relatively distinct C_2_H_2_ and CH_4_, it is still very challenging to separate C_2_H_2_ and CO_2_ due to their similar polarity, molecular shape and geometrical dimensions (3.32 × 3.34 × 5.70 Å^3^ for C_2_H_2_ vs. 3.18 × 3.33 × 5.36 Å^3^ for CO_2_) [32,33,34,35]. Furthermore, most MOFs are sensitive to moisture, which renders them difficult to use under practical conditions where they are exposed to humidity [36]. Therefore, it is urgent to develop water and thermally stable MOFs to facilitate the application of MOFs in gas separation. Besides, another important question that most chemists have neglected is that whether the MOFs can be synthesized on a large scale while maintaining the separation performance [37]. While a few hundred milligram samples are enough for characterization and property measurement in the laboratory, one-pot gram scale synthesis is necessary for practical use.

Herein, we would like to report a highly water and thermal stable MOF Zn_2_(Pydc)(Ata)_2_ (**1**, H_2_Pydc = 3,5-pyridinedicarboxylic acid; HAta = 3-amino-1,2,4-triazole) that is constructed by inexpensive commercially available ligands and zinc ions for efficient separation of C_2_H_2_ from CH_4_ and CO_2_. In our work, **1** was synthesized in a one-pot reaction in a flask with production of 8.6 g. PXRD patterns confirmed its pure phase with identical structures to that of small-scale synthesis in a autoclave. Static single component gas adsorption isotherms showed that **1** could take up 54.9 cm^3^/g of C_2_H_2_ under 278 K and 1 bar, but only 43.1 cm^3^/g of CO_2_ and 27.8 cm^3^/g of CH_4_ under the same conditions. The selectivities calculated by ideal absorbed solution theory (IAST) were 5.1 and 21.5 for equimolar C_2_H_2_/CO_2_ and C_2_H_2_/CH_4_ mixtures, respectively. To confirm the practical separation performance of C_2_H_2_/CO_2_, column breakthrough experiments were carried out for a C_2_H_2_/CO_2_ (50/50) mixture. CO_2_ broke out from the bed packed with **1** at 79 min while C_2_H_2_ was retained in the column for 116 min, indicating the good dynamic separation performance of **1** for C_2_H_2_/CO_2_.

## 2. Results and Discussion

**Scheme 1:**
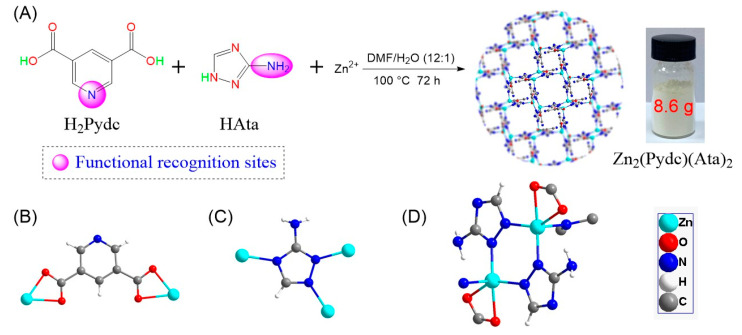
(**A**) Synthetic route towards Zn_2_(Pydc)(Ata)_2_. (**B**) Pydc^2−^ coordination mode. (**C**) Ata^−^ coordination mode. (**D**) Zn^2+^ coordination environment.

The synthesis and crystal structure of Zn_2_(Pydc)(Ata)_2_ (**1**) was first reported by Sun et al. [38]. Compound **1** features uncoordinated pyridyl and amino groups on the pore surface that can serve as recognition sites for interactions with guest molecules and is highly stable up to 400 °C and tolerant of acidic (pH ≥ 2) and basic (pH ≤ 14) aqueous solution conditions, which attracted our interest for the investigation of its application for selective gas separation. In the literature, **1** was synthesized on a very small scale (0.5 mmol) and the yield was specified. Although the stoichiometric ratio of Zn^2+^:Pydc^2−^:Ata^−^ in **1** is 2:1:2, the authors used a substrate ratio of 1:1:1 for the reaction. During the scale-up synthesis (34 mmol) of **1**, we found that the reported 1:1:1 substrate ratio was not reliable, as it led to a large amount of residual H_2_Pydc and a huge reduction of yield. Once we used a Zn(NO_3_)_2_·6H_2_O:H_2_Pydc:HAta = 2:1:2 ratio, no H_2_Pydc residue was observed and the yield was increased to 89% (Scheme 1A). The purity was confirmed by comparing the PXRD patterns of the as-synthesized powder and the one simulated from the single crystal structure (Figure S2). The single crystal structure of **1** revealed that it belongs to the tetragonal space group I4/m, exhibiting a 3D microporous framework. Two carboxylate groups from Pydc^2−^ are paired with two different Zn^2+^ ions, leaving the N sites free (Scheme 1B). For Ata^−^, all three N atoms of the triazole ring are coordinated to three different Zn^2+^ ions while the −NH_2_ group is free (Scheme 1C). Looking at the coordination environment of the Zn^2+^ ion, one can find that a single Zn^2+^ ion is coordinated to two oxygen atoms and three nitrogen atoms (Scheme 1D).

**Figure 1:**
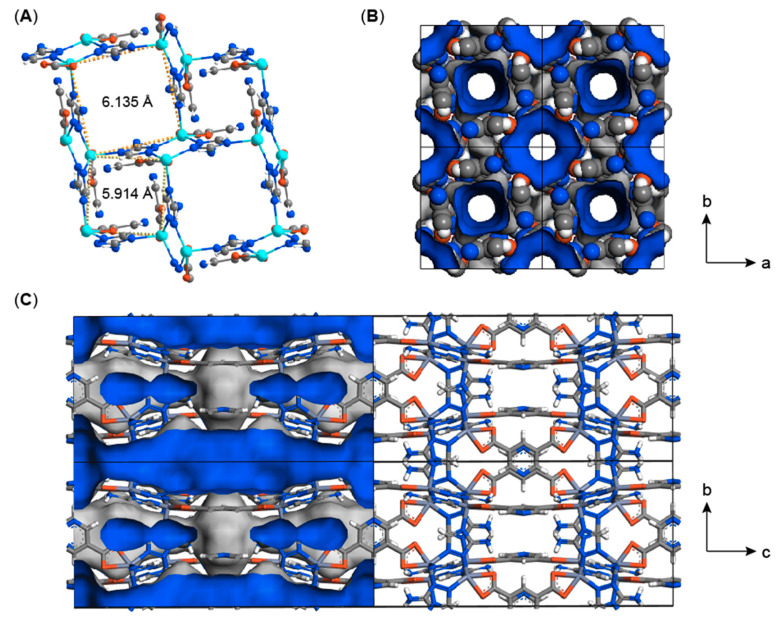
(**A**) Structure of **1** showing the 1D channel with two different dimensions. (**B**,**C**) Connolly isosurface mapping on **1**.

Along the c axis, **1** features two different 1D channels characterized by Zn⋯Zn distances of 6.135 and 5.914 Å (Figure 1A). However, the small channels are not accessible by guest molecules if the flexibility of the ligands is not considered, as indicated by the Connolly surface analysis which gives a Connolly radius of 1.2 Å (Figure 1B). The average diameter of the large channels decorated with electronegative nitrogen sites is 3.6 Å (Figure 1B,C), which is slightly larger than the kinetic diameter of C_2_H_2_ (3.3 Å), CO_2_ (3.3 Å) but slightly smaller than that of CH_4_ (3.8 Å), thus suggesting its use a as a potential material for selective C_2_H_2_/CO_2_ and C_2_H_2_/CH_4_.

**Figure 2:**
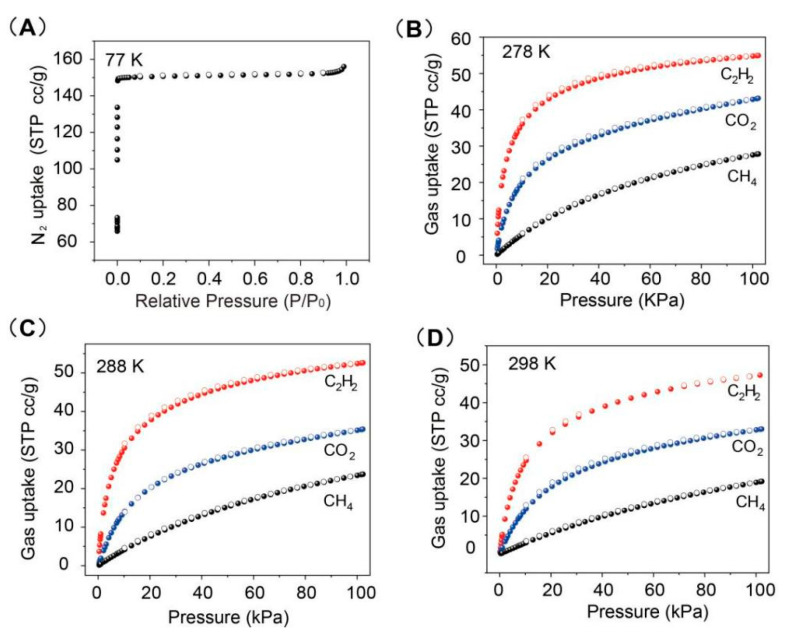
(**A**) N_2_ adsorption/desorption isotherms in **1** at 77 K. (**B**–**D**) C_2_H_2_, CO_2_, and CH_4_ adsorption/desorption isotherms in **1** at 278 K, 288 K, and 298 K.

**Figure 3:**
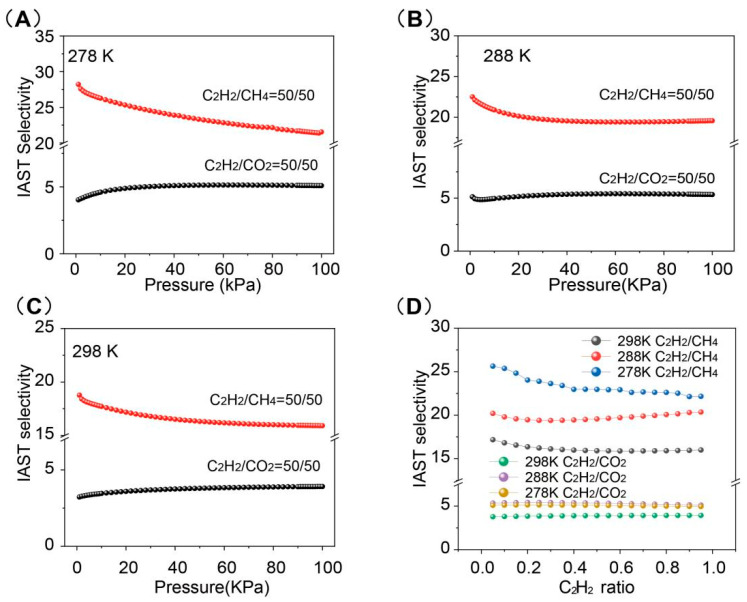
(**A**–**C**) IAST selectivities for equimolar C_2_H_2_/CO_2_ and C_2_H_2_/CH_4_ mixture in **1** at 278, 288, and 298 K. (**D**) IAST selectivities for C_2_H_2_/CO_2_ and C_2_H_2_/CH_4_ mixtures in **1** under 100 kPa with different C_2_H_2_ ratio at 278, 288, and 298 K.

To explore the separation performance of **1**, N_2_ adsorption and desorption isotherms were first collected at 77 K after activation of **1** under vacuum at 200 °C for 12 h. Figure 2A shows that **1** could take up 153 mL/g of N_2_ at P/P_0_ = 0.95 and the adsorption isotherm belonged to typical type I. The pore volume and Brunauer-Emmett-Teller (BET) surface area were calculated to be 0.24 cm^3^/g and 636 m^2^/g, respectively, close to reported values for crystals [38]. Then, C_2_H_2_, CO_2_, and CH_4_ adsorption/desorption isotherms in **1** at 278 K, 288 K, and 298 K were collected, which all exhibited type I isotherms with negligible hysteresis (Figure 2B–D). The capacities of C_2_H_2_, CO_2_, and CH_4_ in **1** at 278 K were 54.9, 43.1, and 27.8 mL/g, respectively, which decreased to 52.6, 35.3, and 23.7 mL/g at 288 K and 47.2, 33.0, and 19.1 mL/g at 298 K. The isotherms were fitted using the dual-site Langmuir–Freundlich equation (Tables S1–S3), and the IAST (ideal absorbed solution theory) selectivities for equimolar C_2_H_2_/CO_2_ and C_2_H_2_/CH_4_ mixtures were calculated and shown in Figure 3A–C. For the C_2_H_2_/CO_2_ mixture, the IAST selectivities were 5.1, 5.3, and 3.9 under 278, 288, and 298 K at 100 kPa, which are comparable to those of many popular MOFs such as BSF-1 (3.3, 298 K) [39], ZJNU-109 (3.6, 288 K) [40], BSF-2 (5.1, 298 K) [41], UTSA-68 (3.4, 298 K) [42] as well as many others shown in Table 1. The IAST selectivities for the C_2_H_2_/CH_4_ mixture were much higher and the values were 21.5, 19.6, and 15.9 under 278, 288, and 298 K at 100 kPa, respectively. These good selectivities of C_2_H_2_ over CO_2_ and CH_4_ originate from the higher affinity of **1** towards C_2_H_2_ by N⋯H-C hydrogen bonding. The IAST selectivities for C_2_H_2_/CO_2_ and C_2_H_2_/CH_4_ mixtures in **1** under 100 kPa with different C_2_H_2_ ratios were also calculated, which indicated that the composition had a larger influence on the C_2_H_2_/CH_4_ mixture, but whether the influence was positive or negative depended on the temperature. At 298 and 278 K, the increasing C_2_H_2_ ratio led to decreased selectivity while it decreased first and increased afterwards with the increase of the C_2_H_2_ ratio at 288 K. The isosteric heats of adsorption (Q_st_) were calculated using the Clausius–Clapeyron equation.

**Figure 4:**
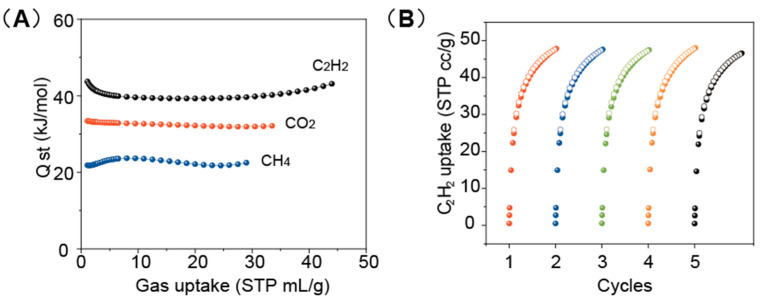
(**A**) Q_st_ for C_2_H_2_, CO_2_ and CH_4_ in **1**. (**B**) Cycling adsorption-desorption experiments. Before every measurement, **1** was regenerated at 200 (1st), 150 (2nd), 100 (3rd) 50 (4th), 25 (5th) °C, respectively, for 30 min.

The Q_st_ values for C_2_H_2_, CO_2_, and CH_4_ at a near-zero loading in **1** were calculated to be 43.1, 32.1, and 22.5 kJ mol^−1^, respectively (Figure 4A). These values were consistent with the adsorption isotherms showing that **1** accommodated C_2_H_2_ more favorably than CO_2_ and CH_4_.

To investigate its recyclability as well as regeneration conditions, cycling C_2_H_2_ adsorption-desorption experiments were conducted on **1**. Figure 4B shows that **1** could be easily regenerated under vacuum at a mild temperature for 30 min. In detail, complete capacity could be realized under exposure to vacuum at temperatures above 50 °C for 30 min while >98% uptake was achieved under vacuum regeneration conditions at 25 °C for 30 min.

**Figure 5:**
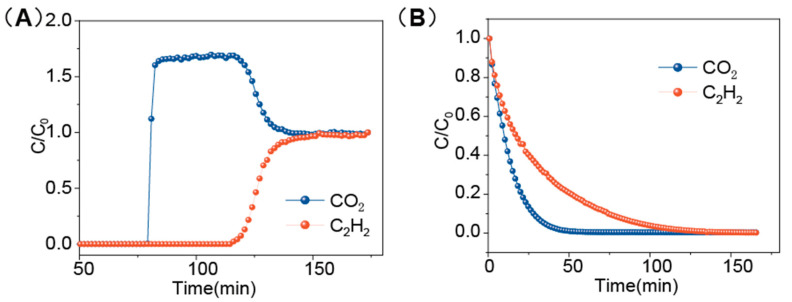
(**A**) Column breakthrough experiments of C_2_H_2_/CO_2_ separation in **1** with a total flowrate of 2 mL/min at 25 °C. (**B**) Regeneration of **1** using an Ar purge of at 100 °C with a flow rate of 3 mL/min.

To further evaluate the dynamic separation of C_2_H_2_/CO_2_ gas mixture, breakthrough experiments were conducted in which an equimolar C_2_H_2_/CO_2_ mixture was flowed over a packed bed of **1** with a total flow of 2 mL/min at 298 K. Figure 5A shows the breakthrough curves of **1** for C_2_H_2_/CO_2_. Compared with C_2_H_2_, CO_2_ eluted first, which could be explained by the favorable affinity of **1** for C_2_H_2_, in agreement with the single-component adsorption isotherms in Figure 2D. The typical roll-up of the CO_2_ curves also revealed the weaker affinity of **1** for CO_2_. The regeneration of **1** with Ar purge was also studied. At 100 °C and with a Ar flowrate of 3 mL/min, nearly all of the adsorbed C_2_H_2_ and CO_2_ can be blown out within 3 h. These experiments confirmed the excellent potential of **1** for practical separation of C_2_H_2_/CO_2_ mixtures as well as the facile regeneration conditions of the material for consecutive use.

## 3. Materials and Methods

### 3.1. Materials

All the materials were used as received without further purification. Zinc nitrate hexahydrate [Zn(NO_3_)_2_·6H_2_O], (98% purity) was purchased from Sinopharm (Shanghai, China). 3,5-Pyridinedicarboxylic acid [H_2_Pydc] (98% purity) and 3-amino-1,2,4-triazole [HAta] (99% purity) were purchased from Energy Chemical (Shanghai, China). N,N-Dimethylformamide [HCON(CH_3_)_2_] (99.5% purity) was purchased from Chinasun Speciality Products Co. (Jinhua, China).

### 3.2. Synthesis of Zn_2_(Pydc)(Ata)_2_ 1

The method for the small scale synthesis of **1** in a autoclave can be found in reference [38] and is summarized here. To a 25 mL autoclave were added 148.7 mg (0.5 mmol) of Zn(NO_3_)_2_·6H_2_O, 42.0 mg (0.5 mmol) of HAta, 83.6 mg (0.5 mmol) of H_2_Pydc, followed by a stir bar. Then 12 mL of DMF and 1 mL of H_2_O were added. The mixture was stirred under room temperature for 10 min whereupon almost all of the solid was dissolved. Then the stir bar was removed and the autoclave was heated to 100 °C and kept at this temperature for 72 h. When cooled to room temperature, slightly yellow crystals were obtained. Then the crystals were collected by filtration, washed with DMF (3 mL × 2) and dried under vacuum at 60 °C for 24 h. According to [38], the obtained solid product has a composition of Zn_2_(Pydc)(Ata)_2_·DMF·2H_2_O. Only by heating at 200 °C under high vacuum can the solvent guest molecules in the pores be removed.

For the 1 g synthesis of Zn_2_(Pydc)(Ata)_2_ the following procedure was performed: A 250 mL round-bottomed flask was charged with Zn(NO_3_)_2_·6H_2_O (1.785 g, 6 mmol), HAta (504.5 mg, 6 mmol) and H_2_Pydc (1.003 g, 6 mmol) and a stir bar. Then 144 mL of DMF and 12 mL of H_2_O were added and the mixture was stirred at 25 °C for about 10 min. Then, the flask bottle was equipped with a condenser, the stirring bar was taken out and the temperature of the oil bath was raised to 100 °C and the heating continued for 72 h. After that, the oil bath was removed, and the round-bottomed flask was allowed to cool to ambient temperature. The resulting powder was collected by filtration, washed with DMF (30 mL), and dried under vacuum at 60 °C for 24 h. The weight after workup was 1.0 g with a yield of 29% based on Zn(NO_3_)_2_·6H_2_O.

For the 10 g synthesis of Zn_2_(Pydc)(Ata)_2_ without increasing the solvent amount a 250 mL round-bottom flask was charged with Zn(NO_3_)_2_·6H_2_O (10.11 g, 34 mmol), HAta (2.86 g, 34 mmol) and H_2_Pydc (5.68 g, 34 mmol) and a stir bar. 144 mL of DMF and 12 mL of H_2_O were added, and the mixture was stirred at 25 °C for about 10 min. Then, the temperature of the oil bath was raised to 100 °C. The flask was equipped with a condenser and the heating was continued for 72 h. The stir bar was not removed for improving the reaction efficiency as the substrate concentrations on this scale were much higher. After that, the oil bath was removed and the flask was allowed to cool to ambient temperature. The resulting powder was collected by filtration and washed with DMF (15 mL × 6). During the washing process, two different kinds of solid with different density were observed and separated. One was white (lower density) and the other was slightly yellow (higher density). After analysis, the white one was found to be H_2_Pydc, while the slightly yellow one was product with > 95% purity (see Figure S2). Both solids were dried under vacuum at 60 °C for 24 h. The weights of recovered H_2_Pydc and yellow product were 2.96 g and 4.33 g, respectively. The yield was 45% based on Zn(NO_3_)_2_·6H_2_O.

Using the improved ratio of starting materials an optimized 10 g synthesis of Zn_2_(Pydc)(Ata)_2_ was performed as follows: A 250 mL round-bottomed flask was charged with Zn(NO_3_)_2_·6H_2_O (10.11 g, 34 mmol), HAta (2.86 g, 34 mmol) and H_2_Pydc (2.84 g, 17 mmol) and a stir bar. 144 mL of DMF and 12 mL of H_2_O were added, and the mixture was stirred at 25 °C for about 10 min. Then, the flask was equipped with a condenser, the temperature of the oil bath was raised to 100 °C and the heating was continued for 72 h. After that, the oil bath was removed, and the flask was allowed to cool to ambient temperature. The resulting powder was collected by filtration, washed with DMF (15 mL × 6). and dried under vacuum at 60 °C for 24 h. The weight after workup was 8.62 g with a yield of 89% based on Zn(NO_3_)_2_·6H_2_O.

### 3.3. Characterization

Powder X-ray diffraction (PXRD) data were collected on an AXS D8-Advance diffractometer (Bruker, Ettlingen, Germany) (Cu Kαλ = 1.540598 Ǻ) with an operating power of 40 KV, 30 mA and a scan speed of 4.0°/min. The range of 2θ was from 5° to 50°.

### 3.4. Adsorption Measurements

The gas adsorption measurements were performed on an Autosorb iQ instrument (Quantachrome, Florida, USA). Before gas adsorption measurements, fresh **1** was evacuated at 200 °C for 12 h until the pressure dropped below 7 μmHg. The adsorption isotherms of C_2_H_2_, CO_2_, and CH_4_ were all collected at 278, 288, and 298 K on activated samples.

### 3.5. Calculation for Adsorption Selectivity and Isosteric Heat of Adsorption

The adsorption isotherms in **1** were fitted using a dual-site Langmuir-Freundlich model:

(1) $$q=q_{A,\;\mathrm{sat}}\frac{b_{A}p^{v_{A}}}{1+b_{A}p^{v_{A}}}+{\;q}_{B,\;\mathrm{sat}}\frac{b_{B}p^{v_{B}}}{1+b_{B}p^{v_{B}}}$$

Here, p (unit: kPa) is the pressure of the bulk gas at equilibrium with the adsorbed phase, q (unit: L kg^−1^) is the adsorbed gas volume per mass of adsorbent, q_A,sat_ an q_B,sat_ (unit: L kg^−1^) are the saturation capacities of site A and B, b_A_ and b_B_ (unit: kPa^−^^v^) are the affinity coefficients of site A and B, and v_A_ and v_B_ represent the deviations from an ideal homogeneous surface.

The isosteric heat of adsorption, Q_st_, is calculated based on the Clausius-Clapeyron equation:

(2) $$Q_{\mathrm{st}}={\;\mathrm{RT}}^{2\;}{\left(\frac{\partial \ln p}{\partial T}\right)}_{q}$$

The IAST adsorption selectivity for two gases is defined as:

(3) $$S_{\mathrm{ads}}=\;\frac{q_{1}/q_{2}}{p_{1}/p_{2}}$$

where q_1_, and q_2_ are the equilibrium gas uptake from the adsorbed phase with partial pressures p_1_, and p_2_

### 3.6. Breakthrough Experiments

The breakthrough experiment was conducted at 298 K on a self-constructed separation setup equipped with a stainless steel column (Φ 4.6 mm × 100 mm). The weight of activated **1** packed in the column was 1.6913 g. The column packed with sample was first purged with a Ar flow (10 mL min^−1^) for 12 h at 100 °C for activation. A C_2_H_2_/CO_2_ = 1/1 (*v*/*v*) gas mixture was then introduced at 2 mL min^−1^. The flow rates of gases were regulated by mass flow controllers and outlet gas concentration was monitored by gas chromatography (GC-9860-5CNJ, Hope, Nanjing, China) using a thermal conductivity detector (TCD) with a detection limit of 100 ppm). After the breakthrough experiment, the column was regenerated with an Ar flow (3 mL min^−1^) at 100 °C for 5 h.

## 4. Conclusions

In conclusion, a large-scale synthetic route to Zn_2_(Pydc)(Ata)_2_ (**1**), a highly water and thermally stable MOF, was reported. The synthesized material was used in the separation of C_2_H_2_ from CH_4_ and CO_2_ with relatively high IAST selectivities for equimolar C_2_H_2_/CO_2_ (5.1) and C_2_H_2_/CH_4_ (21.5) gas mixtures, respectively, comparable to those of many popular MOFs. The practical separation performance for C_2_H_2_/CO_2_ mixture was confirmed by dynamic breakthrough experiments. In addition, **1** can be easily regenerated by vacuum or Ar purging, underscoring its potential use for C_2_H_2_/CO_2_ (5.1) and C_2_H_2_/CH_4_ (21.5) separation in industry.

## Figures and Tables

**Table 1:** Comparison of the gas adsorption performance of **1** and other MOFs.

	Uptake (cm^3^ g^−1^) (298 K, 100 kPa)			IAST Selectivity (298K, 100 kPa)		Ref
	C_2_H_2_	CO_2_	CH_4_	C_2_H_2_/CO_2_	C_2_H_2_/CH_4_	
FJI-C3	43.6	--	11.6	--	14.6	[43]
ZJNU-27	93.7	79.3	26.0	--	16.6	[44]
USTA-36	58.6 ^a^	--	13.2 ^a^	--	13.8 ^a^	[45]
[Co_3_(L)(OH)_2_(H_2_O)]·2DMF·2H_2_O	107.3	62.0	17.7	--	13	[46]
QMOF-1	41.5	24.6	3.9	--	13.5	[47]
FJI-H21	92.4	--	7.10	--	16.3	[48]
JNU-1	63	51	--	3	--	[49]
JXNU-5a	55.9	34.8	--	5	--	[50]
UTSA-68	70.1 ^a^	39.6 ^a^	--	4 ^a^	--	[42]
FJU-36a	52.2 ^a^	35.5 ^a^	10.5 ^a^	2.8 ^a^	17.7 ^a^	[51]
BSF-1	52.5	39.7	10.5	3.3	46.9	[39]
BSF-2	41.5	29.7	5.4	5.1	324	[41]
BSF-3	81.8	47.3	13.4	16.3	205	[6]
ZNU-1 (BSF-9)	76.3	38.1	--	56.6	--	[52]
SNNU-63	91.1	43.7	10.3	3.3	12.9	[53]
ZJNU-109	104.6	60.0	14.3	3.8	21.6	[40]
Zn_2_(Pydc)(Ata)_2_	52.6	35.3	23.6	3.9	15.9	This work

^a^ at 296 K.

## Data Availability

The data that support the findings of this study are available in the Supplementary Materials of this article or from the corresponding author upon reasonable request.

## Supplementary Materials

The following are available online. Figure S1: Photographs illustrating the reaction process, Figure S2: PXRD patterns comparison, Table S1: Langmuir-Freundlich parameters fit for C_2_H_2_, CO_2_, and CH_4_ in Zn_2_(Pydc)(Ata)_2_ at 298 K, Table S2: Langmuir-Freundlich parameters fit for C_2_H_2_, CO_2_, and CH_4_ in Zn_2_(Pydc)(Ata)_2_ at 288 K, Table S3: Langmuir-Freundlich parameters fit for C_2_H_2_, CO_2_, and CH_4_ in Zn_2_(Pydc)(Ata)_2_ at 278 K.

## Sample Availability

Samples of the compounds are not available from the authors.
