# Supplementary material for: Gram-Scale Synthesis of an Ultrastable Microporous Metal-Organic Framework for Efficient Adsorptive Separation of C2H2/CO2 and C2H2/CH4
Source: Molecules. 2021 Aug 24;26(17):5121. doi: 10.3390/molecules26175121 (PMC8433756; doi:10.3390/molecules26175121)

Gram-scale synthesis of an ultrastable microporous metal-organic framework for efficient adsorptive separation of C_2H_2/CO_2 and C_2H_2/CH_4

Nuo Xu[1†], Yunjia Jiang[1†], Wanqi Sun[1], Jiahao Li[1], Lingyao Wang[1], Yujie Jin[2], Yuanbin Zhang[1*], Dongmei Wang,[1*] and Simon Duttwyler[2*]

[1] Key Laboratory of the Ministry of Education for Advanced Catalysis Materials, College of Chemistry and Life Sciences, Zhejiang Normal University, Jinhua 321004, P.R. China

* Correspondence: ybzhang@zjnu.edu.cn, dmwang@zjnu.edu.cn

[2] Department of Chemistry, Zhejiang University 38 Zheda Road, 310027 Hangzhou, P. R. China

* Correspondence: duttwyler@zju.edu.cn

† These authors contributed equally to this work.

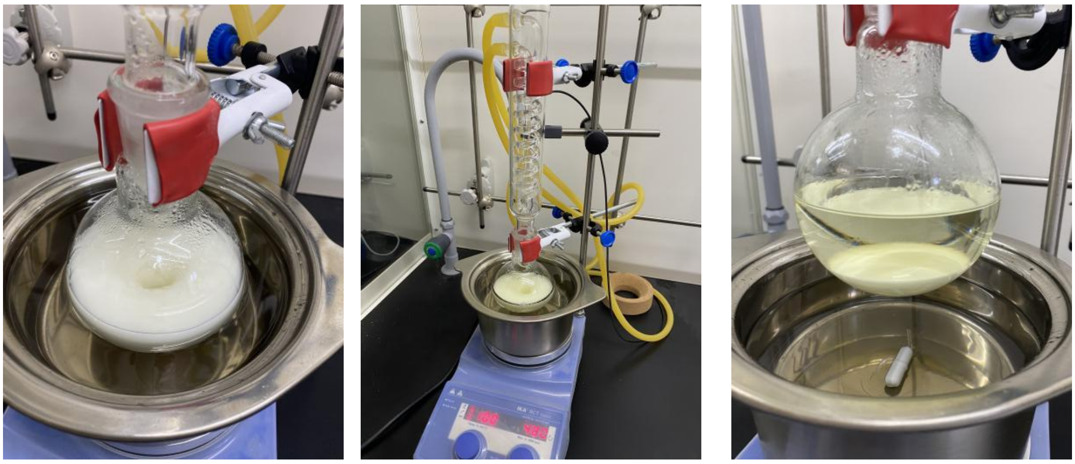

Figure S1. Photographs illustrating the reaction process. At the beginning, many white solid existed in the suspension (Left). After several hours, the white color turned to slightly yellow, indicating the forming of $Zn_2(Pydc)(Ata)_2$ (Middle). After 72 h reaction, the stirring and heating was stopped and a lot of slightly yellow solid was at the bottom of the flask.

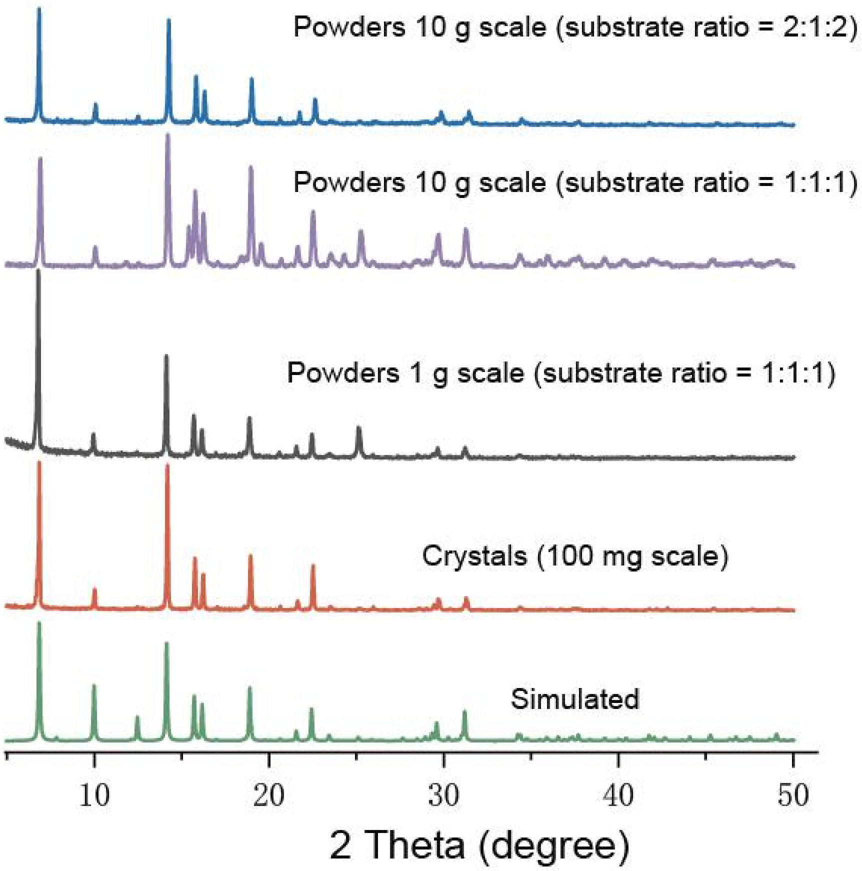

Figure S2. PXRD patterns comparison. The reactions under 10 g scale should be conducted under the correct substrate ratio of 2:1:1. Otherwise, the product is under low purity. However, for reactions below 1 g scale, the substrate ratio of of 1:1:1 is also fine to provide pure product.

Table S1. Langmuir - Freundlich parameters fit for C_2H_2, CO_2 , and CH_4 in $Zn_2(Pydc)(Ata)_2$ at 298 K.

	Site A			Site B			correlation Coefficient (R)
	$q_{A,sat}$ (L kg^{-1})	b_A (kPa^{-vA})	v_A	$q_{B,sat}$ (L kg^{-1})	b_B (kPa^{-vB})	v_B	
C_2H_2	27.661417	0.0161344	0.9530627	33.244053	0.1584683	1.0233041	0.99999925
CO_2	44.556701	0.0024656	0.9490441	29.930424	0.054576	1.010206	0.99999851
CH_4	8.1063163	0.0199379	1.0395496	41.203101	0.0027782	1.1141678	0.99999883

Table S2. Langmuir - Freundlich parameters fit for C_2H_2, CO_2 , and CH_4 in $Zn_2(Pydc)(Ata)_2$ at 288 K.

	Site A			Site B			correlation Coefficient (R)
	$q_{A,sat}$ (L kg^{-1})	b_A (kPa^{-vA})	v_A	$q_{B,sat}$ (L kg^{-1})	b_B (kPa^{-vB})	v_B	
C_2H_2	39.576769	0.0525183	0.779551	27.146765	0.285925	1.0598443	0.99999893
CO_2	101.50684	0.0017424	0.9044426	27.224484	0.0674386	1.0959653	0.99999722
CH_4	50.124832	0.0086188	1	0.1820475	0.0476059	1.9947229	0.99999756

Table S3. Langmuir - Freundlich parameters fit for C_2H_2, CO_2 , and CH_4 in $Zn_2(Pydc)(Ata)_2$ at 278 K.

	Site A			Site B			correlation Coefficient (R)
	$q_{A,sat}$ (L kg^{-1})	b_A (kPa^{-vA})	v_A	$q_{B,sat}$ (L kg^{-1})	b_B (kPa^{-vB})	v_B	
C_2H_2	41.469989	0.0680531	0.7751018	30.83269	0.4670811	1.0363508	0.99999245
CO_2	27.801741	0.1472299	1.0181694	68.296381	0.0068368	0.8369095	0.99999472
CH_4	33.116555	0.0169482	1.0222692	40.418688	0.0014187	1.0415364	0.99999918

Scanning electron microscope (SEM) was carried out on a FEI NOVA NanoSEM 450 instrument. SEM imaging showed the as-synthesized $Zn_2(Pydc)(Ata)_2$ had a block-like particle morphology with average crystalline sizes of 0.5~3 um.

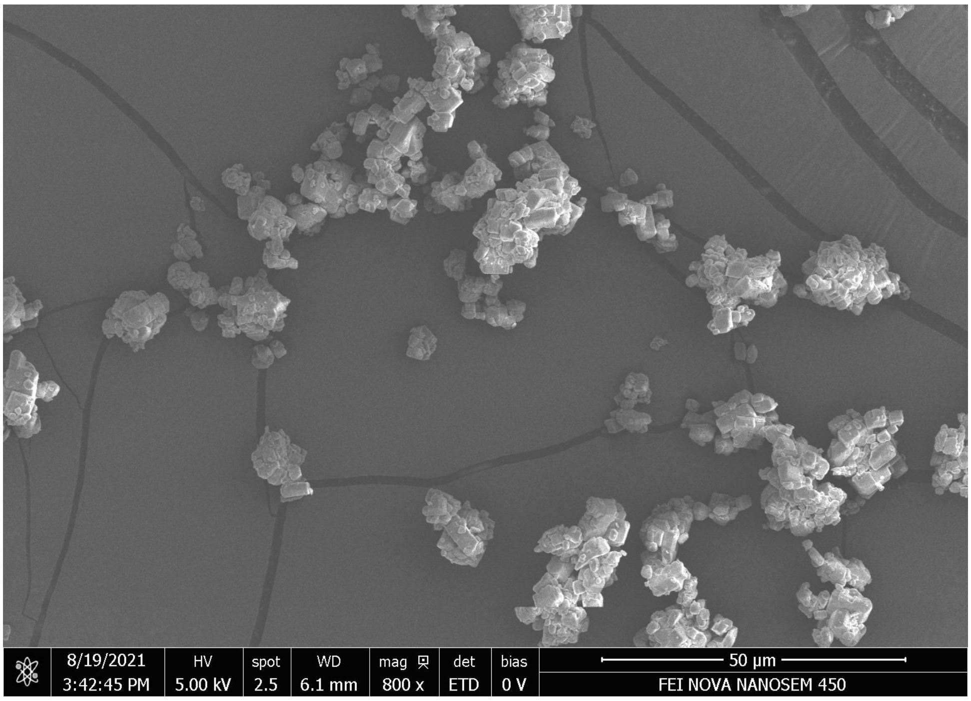

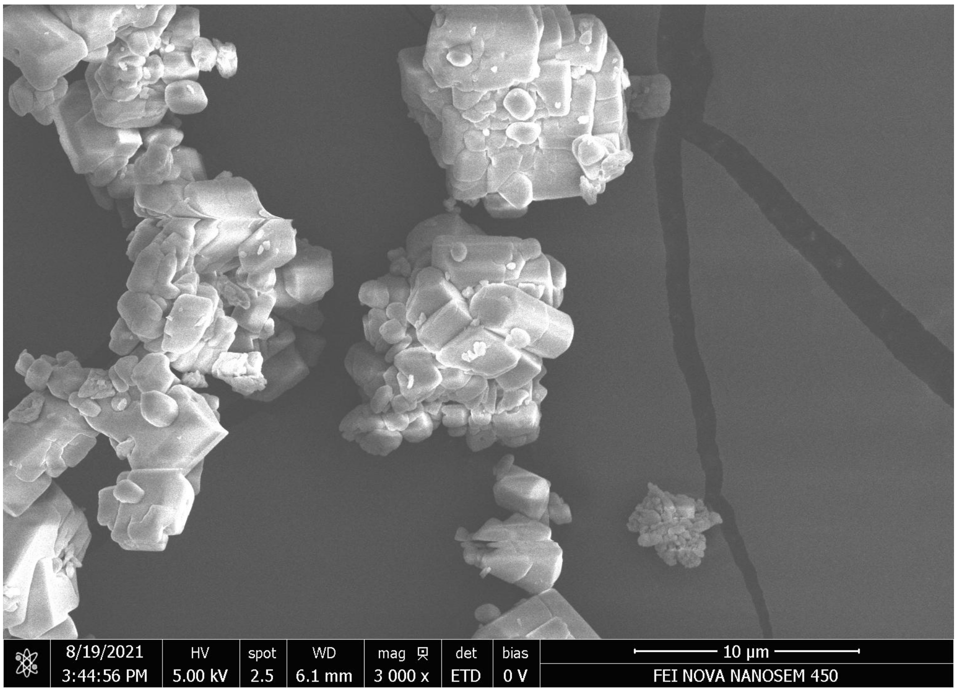

TGA was performed in a NETZSCH STA 449F3 instrument from 30 to 800 °C in N_2 atmosphere at a constant rate of 10 °C/min. The sample was dried in a oven at 120 °C for 12 h before measurement. Thus, the mass loss from RT to 250 °C was different from the values from the literature, which was tested without any pre-treatment. From the TGA curves, $Zn_2(Pydc)(Ata)_2$ was stable up to ~400 °C. This is consistent with the literature.

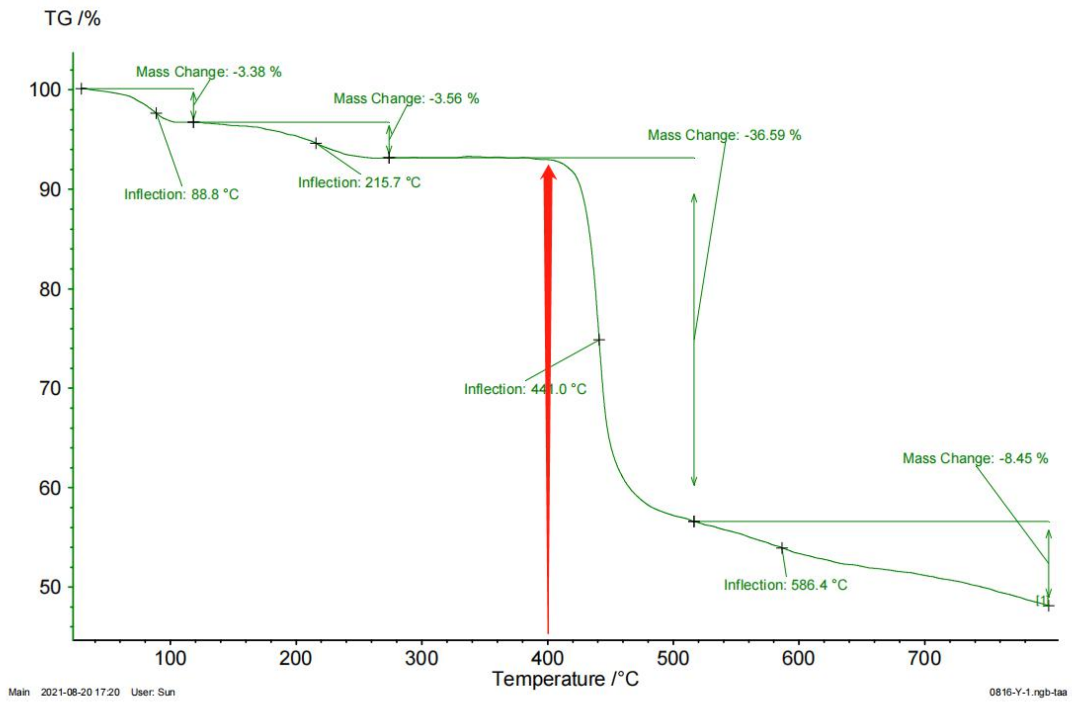

Supplement: Supplementary file 1 [file molecules-26-05121-s001.zip › molecules-1337979-supplementary.pdf]
